# Supplementary material for: Aerobic Capacity and Exercise Mediate Protection Against Hepatic Steatosis via Enhanced Bile Acid Metabolism
Source: Function (Oxf). 2025 Apr 7;6(3):zqaf019. doi: 10.1093/function/zqaf019 (PMC12086534; doi:10.1093/function/zqaf019)
Supplement: zqaf019_Supplemental_File [file zqaf019_supplemental_file.docx]

**Supplemental Table S1.** MRM transition of Bas and deuterium-labeled internal standards

| **Compound** | **MID** | **Q1 m/z** | **Q3 m/z** |
| --- | --- | --- | --- |
| TMCA, TCA | M+0  M+1  M+2  M+3 | 514.2  515.2  516.2  517.2 | 80.0  80.0  80.0  80.0 |
| TCDCA, TDCA | M+0  M+1  M+2  M+3 | 498.2  499.2  500.2  501.2 | 80.0  80.0  80.0  80.0 |
| d9-TCDCA |  | 507.2 | 80.0 |

**Supplemental Table S2.** Anthropometric and energy intake data from HCR/LCR rats on a LFD or HFD for 1-week.

|  | **LCR** | | | | | | **HCR** | | | | | | **P-value** | | |
| --- | --- | --- | --- | --- | --- | --- | --- | --- | --- | --- | --- | --- | --- | --- | --- |
| **Variable** | **LFD** | | | **HFD** | | | **LFD** | | | **HFD** | | | **Strain** | **Diet** | **Strain x Diet** |
| ∆ Body Mass (g) | 8.9 | ± | 1.7 | 26.6 | ± | 2.2 | 7.1 | ± | 2.3 | 15.8 | ± | 1.4*,^ | **0.0003** | **0.001** | **0.027** |
| ∆ Fat Mass (g) | 2.3 | ± | 1.0 | 19 | ± | 2.3*** | 6.2 | ± | 1.5 | 9.7 | ± | 2.2 | 0.155 | **0.001** | **0.001** |
| ∆ Lean Mass (g) | -1.3 | ± | 1.2 | -2.5 | ± | 2.4 | -7.5 | ± | 1.6 | -1.6 | ± | 1.5 | 0.135 | 0.187 | 0.052 |
| Body Mass (g) | 452 | ± | 14.8 | 480 | ± | 15.4 | 350 | ± | 14.1 | 356 | ± | 8.9 | **<0.001** | 0.213 | 0.445 |
| % Fat Mass | 15.9 | ± | 1.0 | 19.7 | ± | 1.1 | 9.7 | ± | 0.5 | 11.6 | ± | 1.0 | **<0.001** | **0.006** | 0.342 |
| % Lean Mass | 76.6 | ± | 0.9 | 73 | ± | 1.1 | 83.1 | ± | 0.5 | 81.1 | ± | 1.1 | **<0.001** | **0.005** | 0.355 |
| Total Energy Intake (kcal) | 418 | ± | 18.6 | 612 | ± | 35.1*** | 460 | ± | 28.6 | 524 | ± | 14.4 | 0.377 | **0.001** | **0.016** |

Values are means ± SEM (n=8); Only rats maintaining access to food prior to sacrifice). *indicates effect of diet within strain (*p<0.05, **p<0.01, ***p<0.001); ^indicates effect of strain within diet (^p<0.05).

**Supplemental Table S3.** Serum metabolic data from HCR/LCR rats on a LFD or HFD for 1-week.

Values are means ± SEM (n=8 per group). *indicates the effect of diet within a strain (*p<0.05, **p<0.01, ***p<0.001); ‡indicates the effect of the fed state within strain (‡p<0.05, ‡‡p<0.01, ‡‡‡p<0.001).

**Supplemental Table S4.** Anthropometric and energy intake data from HCR/LCR rats on a LFD or HFD for 20-weeks.

|  | **LCR** | | | | | | **HCR** | | | | | | **P-value** | | |
| --- | --- | --- | --- | --- | --- | --- | --- | --- | --- | --- | --- | --- | --- | --- | --- |
| **Variable** | **LFD** | | | **HFD** | | | **LFD** | | | **HFD** | | | **Strain** | **Diet** | **Strain x Diet** |
| ∆ Body Mass (g) | 75.3 | ± | 12.9 | 107.3 | ± | 12.7 | 69.3 | ± | 6.6 | 89.9 | ± | 8.1 | 0.27 | **0.016** | 0.586 |
| Body Mass (g) | 505.5 | ± | 16.5 | 541.0 | ± | 13.0 | 410.5 | ± | 11.9 | 426.0 | ± | 20.7 | **<0.001** | 0.117 | 0.533 |
| % Fat Mass | 19.8 | ± | 2.3 | 25.9 | ± | 1.7 | 16.0 | ± | 1.0 | 18.6 | ± | 1.4 | **0.002** | **0.013** | 0.298 |
| % Lean Mass | 72.7 | ± | 2.1 | 67.0 | ± | 1.7 | 76.1 | ± | 1.1 | 73.9 | ± | 1.3 | **0.003** | **0.018** | 0.285 |
| Weekly Energy Intake (kcal) | 406.3 | ± | 8.3 | 428.8 | ± | 7.1 | 388.5 | ± | 10.2 | 406.7 | ± | 13.8 | 0.058 | 0.052 | 0.833 |

Values are means ± SEM (n=10 per group).

**Supplemental Table S5.** Serum metabolic data from HCR/LCR rats on a LFD or HFD for 20-weeks.

|  | **LCR** | | | | | | | | **HCR** | | | | | | | | **P-value** | | | | | |
| --- | --- | --- | --- | --- | --- | --- | --- | --- | --- | --- | --- | --- | --- | --- | --- | --- | --- | --- | --- | --- | --- | --- |
| **Variable** | **LFD** | | | | **HFD** | | | | **LFD** | | | | **HFD** | | | | **Strain** | | **Diet** | | **Strain x Diet** | |
| ALP (U/L) | 161.1 | ± | 12.9 | 230.9 | | ± | 15.6 | 189.9 | | ± | 13.1 | 231.0 | | ± | 18.5 | 0.348 | | **<0.001** | | 0.351 | |  |
| AST (U/L) | 156.7 | ± | 16.3 | 180.9 | | ± | 13.7 | 169.7 | | ± | 13.6 | 148.1 | | ± | 19.4 | 0.538 | | 0.935 | | 0.159 | |  |
| ALT (U/L) | 87.2 | ± | 11.0 | 113.0 | | ± | 11.9 | 124.9 | | ± | 18.9 | 107.7 | | ± | 22.6 | 0.342 | | 0.800 | | 0.210 | |  |
| Albumin (g/dL) | 3.3 | ± | 0.1 | 3.3 | | ± | 0.0 | 3.3 | | ± | 0.1 | 3.1 | | ± | 0.1 | 0.305 | | 0.137 | | 0.238 | |  |
| Total Protein (g/dL) | 6.0 | ± | 0.1 | 6.0 | | ± | 0.1 | 6.1 | | ± | 0.1 | 5.7 | | ± | 0.1 | 0.418 | | **0.044** | | 0.110 | |  |
| BUN (mg/dL) | 16.5 | ± | 0.6 | 15.3 | | ± | 0.6 | 16.0 | | ± | 0.7 | 14.3 | | ± | 1.1 | 0.342 | | 0.071 | | 0.750 | |  |
| Cholesterol (mg/dL) | 122.6 | ± | 4.8 | 116 | | ± | 4.9 | 97.0 | | ± | 4.5 | 93.3 | | ± | 3.5 | **<0.001** | | 0.255 | | 0.747 | |  |
| Glucose (mg/dL) | 220.4 | ± | 8.5 | 203.3 | | ± | 6 | 247.4 | | ± | 14.7 | 213.9 | | ± | 7.9 | 0.065 | | **0.015** | | 0.411 | |  |
| Triglycerides (mg/dL) | 77.3 | ± | 5.9 | 134.5 | | ± | 7.2** | 137.6 | | ± | 12.0^^ | 109.2 | | ± | 17.2 | 0.135 | | 0.216 | | **<0.001** | |  |
| β-Hydroxybutyrate (mg/dL) | 2.6 | ± | 0.2 | 3.7 | | ± | 0.3 | 2.3 | | ± | 0.1 | 4.0 | | ± | 0.3 | 0.913 | | **<0.001** | | 0.199 | |  |
| NEFA (mEq/L) | 0.26 | ± | 0.04 | 0.55 | | ± | 0.04 | 0.4 | | ± | 0.07 | 0.49 | | ± | 0.07 | 0.474 | | **0.002** | | 0.083 | |  |

Values are means ± SEM (n=10 per group). *indicates effect of diet within strain (*p<0.05, **p<0.01, ***p<0.001); ^indicates effect of strain within diet (^p<0.05, ^^p<0.01, ^^^p<0.001).

**Supplemental Table S6.** Serum bile acid data from HCR/LCR rats on a LFD or HFD for 1-week.

Values are means ± SEM (n=8 per group). ‡indicates the effect of the fed state within a strain (‡p<0.05, ‡‡p<0.01, ‡‡‡p<0.001).

**Supplemental Table S7.** Serum bile acid data from HCR/LCR rats on a LFD or HFD for 20-weeks.

| **(ng/mL)** | **LCR** | | | | | | **HCR** | | | | | | **P-value** | | |
| --- | --- | --- | --- | --- | --- | --- | --- | --- | --- | --- | --- | --- | --- | --- | --- |
|  | **LFD** | | | **HFD** | | | **LFD** | | | **HFD** | | | **Strain** | **Diet** | **Strain x Diet** |
| G-CA | 25.00 | ± | 7.83 | 22.28 | ± | 4.73 | 11.16 | ± | 3.67 | 11.38 | ± | 4.34 | **0.028** | 0.818 | 0.787 |
| G-CDCA | 0.65 | ± | 0.16 | 0.42 | ± | 0.09 | 0.37 | ± | 0.10 | 0.14 | ± | 0.07 | **0.023** | 0.062 | 0.965 |
| G-DCA | 0.59 | ± | 0.39 | 0.38 | ± | 0.06 | 0.07 | ± | 0.04 | 0.12 | ± | 0.05 | 0.056 | 0.688 | 0.581 |
| G-UDCA | 0.14 | ± | 0.05 | 0.05 | ± | 0.03 | 0.05 | ± | 0.04 | 0.03 | ± | 0.03 | 0.152 | 0.231 | 0.629 |
| T-αMCA | 62.16 | ± | 9.57 | 79.38 | ± | 22.60 | 48.16 | ± | 9.96 | 37.05 | ± | 11.86 | 0.060 | 0.834 | 0.336 |
| βMCA | 283.79 | ± | 72.43 | 342.29 | ± | 135.28 | 85.79 | ± | 25.56 | 81.49 | ± | 35.87 | **0.007** | 0.736 | 0.696 |
| T-CA | 944.20 | ± | 187.82 | 1697.34 | ± | 366.09 | 208.66 | ± | 50.32 | 459.47 | ± | 157.16 | **<0.001** | **0.030** | 0.265 |
| T-CDCA | 32.16 | ± | 5.80 | 38.92 | ± | 10.34 | 18.21 | ± | 4.32 | 16.67 | ± | 6.21 | **0.014** | 0.712 | 0.558 |
| T-DCA | 25.57 | ± | 4.31 | 48.23 | ± | 8.12 | 7.52 | ± | 1.87 | 16.26 | ± | 4.86 | **<0.001** | **0.005** | 0.196 |
| T-UDCA | 15.87 | ± | 4.47 | 17.35 | ± | 5.96 | 6.68 | ± | 2.40 | 5.93 | ± | 2.99 | **0.019** | 0.931 | 0.790 |
| αMCA | 9.74 | ± | 6.68 | 3.56 | ± | 1.53 | 6.09 | ± | 4.88 | 3.10 | ± | 1.31 | 0.635 | 0.290 | 0.709 |
| βMCA | 42.66 | ± | 26.76 | 20.01 | ± | 7.08 | 12.99 | ± | 4.55 | 14.67 | ± | 4.12 | 0.225 | 0.464 | 0.397 |
| CA | 91.76 | ± | 80.61 | 79.71 | ± | 49.30 | 32.08 | ± | 26.44 | 26.24 | ± | 13.50 | 0.261 | 0.858 | 0.950 |
| CDCA | 3.54 | ± | 3.81 | 2.11 | ± | 1.86 | 7.67 | ± | 8.12 | 1.91 | ± | 1.24 | 0.674 | 0.442 | 0.643 |
| DCA | 12.59 | ± | 3.54 | 15.58 | ± | 2.05 | 7.31 | ± | 1.25 | 14.38 | ± | 2.77 | 0.212 | 0.056 | 0.431 |
| LCA | 8.50 | ± | 2.82 | 3.30 | ± | 0.94 | 3.17 | ± | 0.85 | 4.10 | ± | 0.63 | 0.160 | 0.185 | 0.059 |
| UDCA | 1.53 | ± | 1.10 | 1.06 | ± | 0.52 | 1.44 | ± | 0.53 | 1.34 | ± | 0.39 | 0.874 | 0.688 | 0.774 |
| G-TOTALS | 26.30 | ± | 8.36 | 23.11 | ± | 4.85 | 11.56 | ± | 3.73 | 11.58 | ± | 4.42 | **0.026** | 0.779 | 0.778 |
| T-TOTALS | 1363.92 | ± | 275.94 | 2223.43 | ± | 533.83 | 374.94 | ± | 90.89 | 616.86 | ± | 215.99 | **<0.001** | 0.096 | 0.345 |
| Unconj TOTALS | 170.31 | ± | 121.71 | 125.33 | ± | 60.55 | 70.76 | ± | 43.92 | 65.75 | ± | 20.38 | 0.277 | 0.731 | 0.783 |
| % G-TOTALS | 1.66 | ± | 0.36 | 1.53 | ± | 0.71 | 2.88 | ± | 1.10 | 2.46 | ± | 0.57 | 0.150 | 0.707 | 0.844 |
| % T-TOTALS | 87.36 | ± | 6.10 | 90.85 | ± | 3.20 | 82.72 | ± | 6.49 | 77.06 | ± | 6.93 | 0.125 | 0.854 | 0.441 |
| % Unconj TOTALS | 10.98 | ± | 5.85 | 7.62 | ± | 2.74 | 14.40 | ± | 5.99 | 20.48 | ± | 6.62 | 0.148 | 0.806 | 0.397 |
| 12-hydroxy/non-hydroxy | 2.38 | ± | 0.18 | 4.85 | ± | 0.53 | 1.56 | ± | 0.23 | 3.04 | ± | 0.30 | **<0.001** | **<0.001** | 0.153 |
| **TOTAL (ng/mL)** | 1560.53 | ± | 274.48 | 2371.87 | ± | 534.21 | 457.25 | ± | 93.92 | 694.18 | ± | 212.12 | **<0.001** | 0.112 | 0.378 |
|  |  |  |  |  |  |  |  |  |  |  |  |  |  |  |  |

Values are means ± SEM (n=10 per group).

**Supplemental Table S8.** Anthropometric, energy intake, and wheel running data from mice on a sedentary and voluntary wheel running mice.

| **Variable** | **SED** | | | **VWR** | | | **P-value** |
| --- | --- | --- | --- | --- | --- | --- | --- |
| ∆ Body Mass (g) | 6.68 | ± | 0.75 | 3.86 | ± | 0.66 | **0.029** |
| ∆ Fat Mass (g) | 6.81 | ± | 0.52 | 3.86 | ± | 0.62 | **0.022** |
| ∆ Lean Mass (g) | -1.86 | ± | 0.37 | 0.35 | ± | 0.51 | **0.026** |
| Body Mass (g) | 36.31 | ± | 0.74 | 32.23 | ± | 1.02 | **0.001** |
| % Fat Mass | 0.28 | ± | 0.01 | 0.19 | ± | 0.02 | **0.039** |
| % Lean Mass | 0.64 | ± | 0.01 | 0.72 | ± | 0.02 | **0.006** |
| Avg Daily Energy Intake (kcal) | 11.78 | ± | 0.21 | 15.46 | ± | 0.42 | **0.001** |
| Avg Daily Running Distance (km) | - |  | - | 10.02 | ± | 0.52 |  |

Values are means ± SEM (n=7-8).

**Supplemental Table S9.** Male liver-specific Cyp7a1 KO anthropometric, energy intake, and wheel running data from sedentary and voluntary wheel running mice.

|  | **Ctrl** | | **LCyp7a1KO** | | **P-Value** | | |
| --- | --- | --- | --- | --- | --- | --- | --- |
|  | **SED** | **VWR** | **SED** | **VWR** | **Genotype** | **Exercise** | **Genotype x Exercise** |
| Body Weight (g) | 32.2 ± 2.2 | 32.3 ± 1.8 | 32.4 ± 1.4 | 34.6 ± 1.4 | 0.489 | 0.509 | 0.544 |
| Δ Body Weight (g) | 0.7 ± 0.4 | 0.8 ± 0.3 | -0.1 ± 0.4 | 0.4 ± 0.3 | 0.074 | 0.439 | 0.596 |
| Δ Fat Mass (g) | 0.6 ± 0.2 | -0.8 ± 0.9 | -1.0 ± 0.6 | -2.0 ± 0.9 | 0.061 | 0.114 | 0.773 |
| Δ Fat Free Mass (g) | -0.1 ± 0.3 | 0.3 ± 0.2 | 0.7 ± 0.4 | 1.2 ± 0.3 | **0.007** | 0.133 | 0.922 |
| Avg Daily Energy Intake (kcal) | 8.9 ± 0.4 | 11.7 ± 0.6 | 10.1 ± 0.4 | 12.5 ± 0.7 | 0.067 | **0.001** | 0.730 |
| Avg. Daily Running Distance (km) | - | 6.3 ± 1.3 | - | 5.6 ± 0.8 | 0.662 | - | - |

Values are means ± SEM (n=6-8).

**Supplemental Table S10.** Female **l**iver-specific Cyp7a1 KO anthropometric, energy intake, and wheel running data from sedentary and voluntary wheel running mice.

|  | **Ctrl** | | **LCyp7a1KO** | | **P-Value** | | |
| --- | --- | --- | --- | --- | --- | --- | --- |
|  | **SED** | **VWR** | **SED** | **VWR** | **Genotype** | **Exercise** | **Genotype x Exercise** |
| Body Weight (g) | 24.4 ± 1.8 | 22.7 ± 0.5 | 24.0 ± 1.3 | 23.5 ± 0.6 | 0.838 | 0.334 | 0.600 |
| Δ Body Weight (g) | 0.4 ± 0.3 | -0.4 ± 0.2 | 0.5 ± 0.2 | 0.5 ± 0.5 | 0.200 | 0.363 | 0.363 |
| Δ Fat Mass (g) | 0.2 ± 0.5 | -1.5 ± 0.9 | 0.5 ± 0.2 | -1.7 ± 0.5 | 0.983 | **0.008** | 0.722 |
| Δ Fat Free Mass (g) | 0.5 ± 0.2 | 0.9 ± 0.2 | -0.1 ± 0.3 | 0.3 ± 0.1 | **0.024** | 0.107 | 0.895 |
| Avg Daily Energy Intake (kcal) | 8.4 ± 0.5 | 10.3 ± 0.6 | 8.3 ± 0.4 | 11.0 ± 0.6 | 0.606 | **0.001** | 0.500 |
| Avg. Daily Running Distance (km) | - | 8.8 ± 0.9 | - | 8.6 ± 0.2 | 0.830 | - | - |

Values are means ± SEM (n=6-8).

**Supplemental Table S11.** Male liver-specific Cyp7a1KO **l**iver bile acid content from sedentary and voluntary wheel-running mice.

|  | **Ctrl** | | **LCyp7a1KO** | | **P-Value** | | |
| --- | --- | --- | --- | --- | --- | --- | --- |
| **(ug/g of liver)** | **SED** | **VWR** | **SED** | **VWR** | **Genotype** | **Exercise** | **Genotype x Exercise** |
| T-αMCA | 2.76 ± 0.46 | 1.20 ± 0.24***** | 0.58 ± 0.15***** | 0.32 ± 0.05**^#^** | **0.001** | **0.001** | **0.016** |
| T-βMCA | 11.11 ± 6.03 | 3.60 ± 0.65 | 4.02 ± 0.90 | 5.84 ± 0.66 | 0.370 | 0.293 | 0.091 |
| T-CA | 32.62 ± 11.53 | 16.28 ± 2.67 | 9.32 ± 2.34 | 11.17 ± 1.63 | **0.013** | 0.184 | 0.099 |
| T-CDCA | 3.39 ± 1.24 | 1.12 ± 0.18 | 0.60 ± 0.17 | 0.47 ± 0.13 | **0.004** | **0.039** | 0.062 |
| T-DCA | 3.26 ± 0.59 | 3.81 ± 0.54 | 1.05 ± 0.29 | 1.07 ± 0.18 | **0.001** | 0.505 | 0.527 |

Values are means ± SEM (n=6-8). *P<0.05 vs Ctrl+Sed group; #P<0.05 vs Ctrl+VWR.

**Supplemental Table S12.** Female liver-specific Cyp7a1KO liver bile acid content from sedentary and voluntary wheel-running mice.

|  | **Ctrl** | | **LCyp7a1KO** | | **P-Value** | | |
| --- | --- | --- | --- | --- | --- | --- | --- |
| **(ug/g of liver)** | **SED** | **VWR** | **SED** | **VWR** | **Genotype** | **Exercise** | **Genotype x Exercise** |
| T-αMCA | 2.01 ± 0.41 | 2.19 ± 0.28 | 0.11 ± 0.03 | 0.88 ± 0.72 | **0.001** | 0.308 | 0.520 |
| T-βMCA | 5.51 ± 1.04 | 9.11 ± 1.62 | 3.77 ± 1.27 | 3.29 ± 0.64 | **0.005** | 0.216 | 0.110 |
| T-CA | 27.61 ± 5.29 | 48.25 ± 7.89 | 11.30 ± 2.70 | 16.58 ± 2.60 | **0.001** | **0.019** | 0.149 |
| T-CDCA | 2.55 ± 0.34 | 2.75 ± 0.40 | 0.21 ± 0.06 | 0.70 ± 0.41 | **0.001** | 0.314 | 0.668 |
| T-DCA | 4.31 ± 0.96 | 7.88 ± 1.64 | 0.36 ± 0.13 | 0.83 ± 0.20 | **0.001** | **0.048** | 0.124 |

Values are means ± SEM (n=6-8).


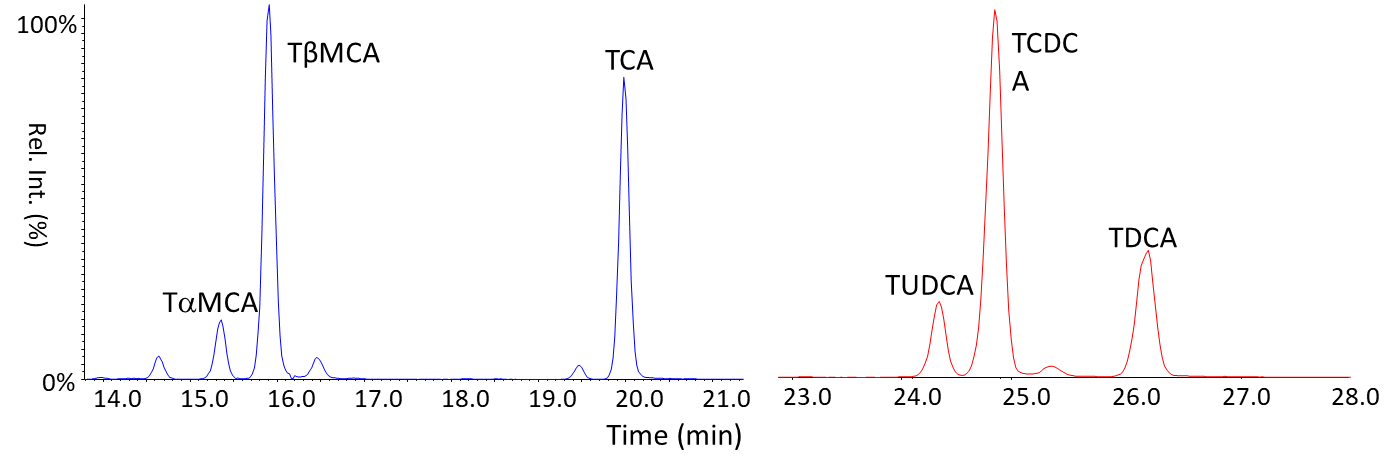


**Supplemental Figure 1.** Representative LC-MS/MS chromatogram of rat liver tissue. Structural isomers, T-αMCA, T-βMCA, and T-CA were completely separated. T-UDCA, T-CDCA, and T-DCA isomers were baseline-separated as well.


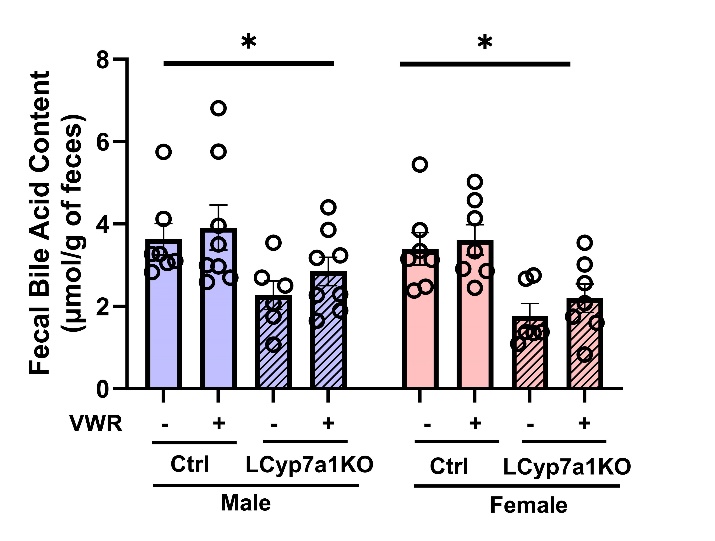

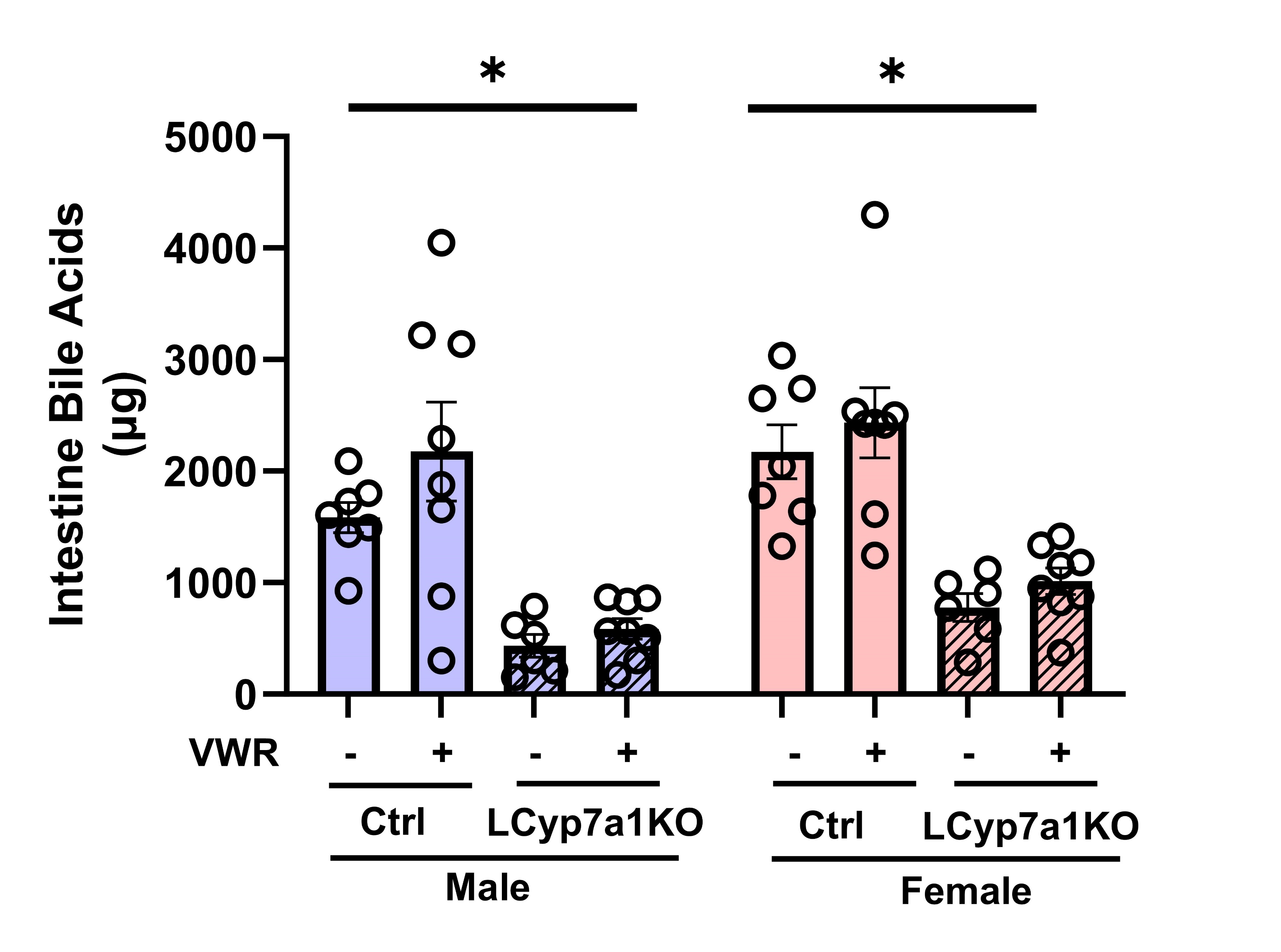

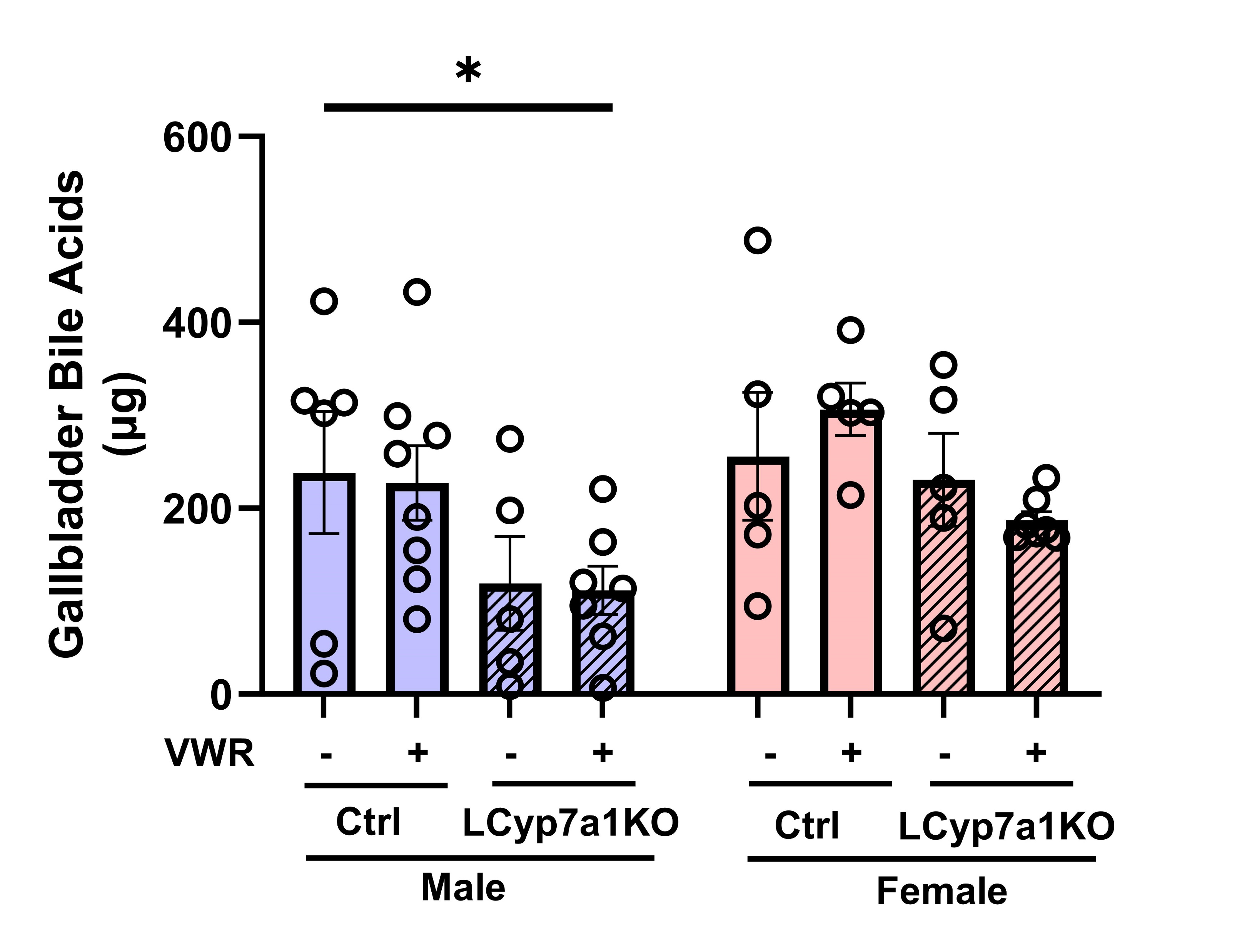

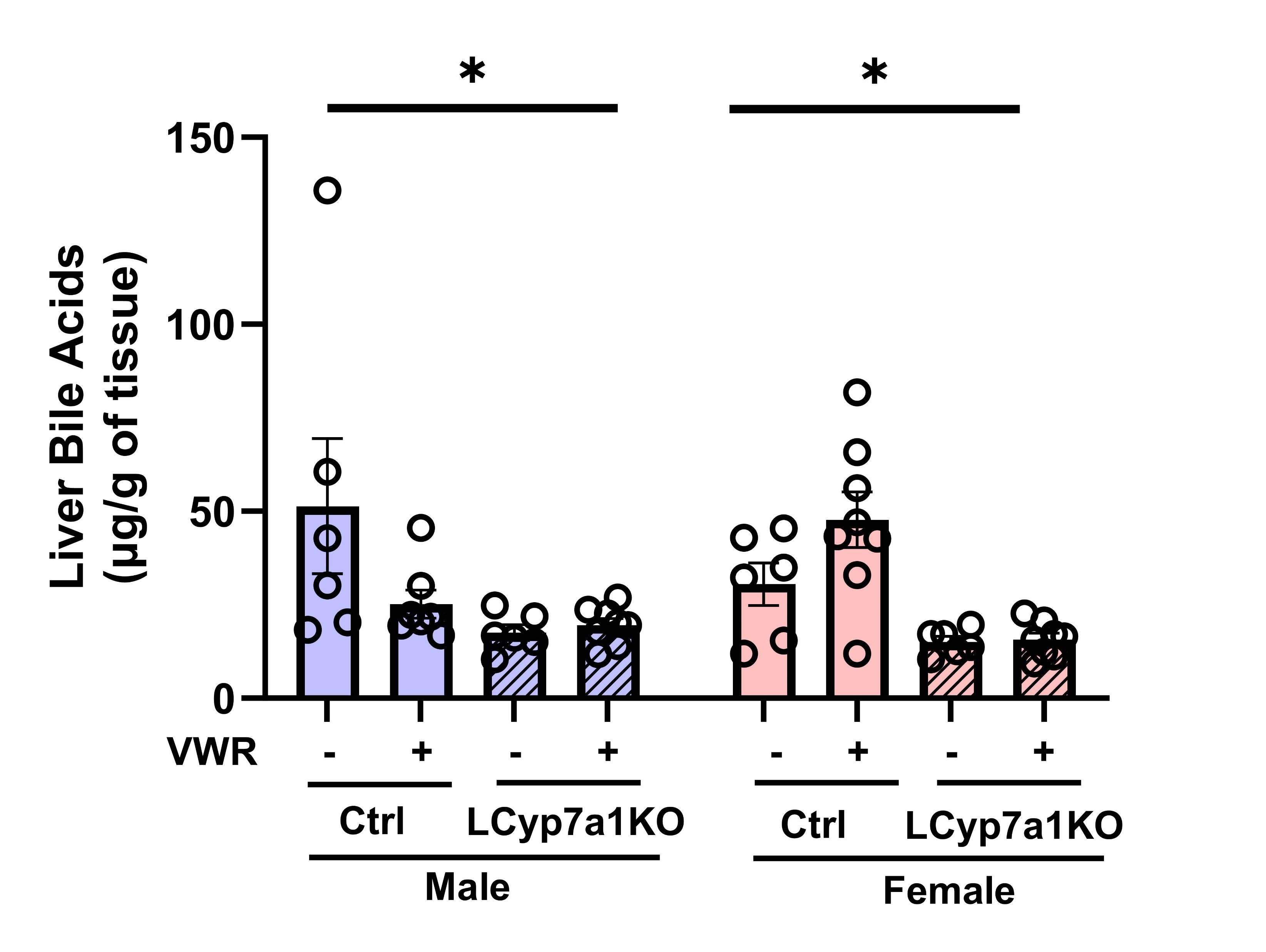


**A.**

**B.**

**C.**

**D.**

**Supplemental Figure 2.** Bile acid content in liver-specific Cyp7a1 knockout mice with VWR. **A.** Liver bile content. **B.** Gallbladder bile acid content. **C.** Intestine bile acid content. **D**. Fecal bile acid content. Data represented as means ± SEM, (n=6-8). *indicates main effect of LCyp7a1 within sex (*P<0.05).
